# Supplementary material for: Systematic review on e-cigarette and its effects on weight gain and adipocytes
Source: PLoS One. 2022 Jul 5;17(7):e0270818. doi: 10.1371/journal.pone.0270818 (PMC9255744; doi:10.1371/journal.pone.0270818)
Supplement: S1 Table — (DOCX) [file pone.0270818.s001.docx]

|  | | | Oh etal. | | Kim et al. | | Hasan et al. | | | Bennett et al. | | Delk et al. | | | Cho et al. | | | Rhoades et al. | | | Wetendorf et al. | | | Strong et al. | | | Morean &Wedel | | | Lanza et al. | | Zagoritti et al. | | | Wang et al. | |
| --- | --- | --- | --- | --- | --- | --- | --- | --- | --- | --- | --- | --- | --- | --- | --- | --- | --- | --- | --- | --- | --- | --- | --- | --- | --- | --- | --- | --- | --- | --- | --- | --- | --- | --- | --- | --- |
| **OHAT risk-of-bias tool** | | |  | | | | | | | | | | | | | | | | | | | | | | | | | | | | | | | | |  |
| 1. Was administered dose or exposure level adequately randomized? | | | NR | | NR | |  | | | NR | | NR | | NR | | | NR | | |  | | | NR | | | NR | | | NR | | NR | | | NR | | |
| 2. Was allocation to study groups adequately concealed? | | | NR | | NR | |  | | | NR | | NR | | NR | | | NR | | |  | | | NR | | | NR | | | NR | | NR | | | NR | | |
| 3. Did selection of study participants result in the appropriate comparison groups? | | |  | |  | | NR | | |  | |  | |  | | |  | | | NR | | |  | | |  | | |  | | NR | | |  | | |
| 4. Did study design or analysis account for important confounding and modifying variables? | | |  | |  | | NR | | |  | |  | |  | | |  | | | NR | | |  | | |  | | |  | | NR | | |  | | |
| 5. Were experimental conditions identical across study group | | | NR | | NR | |  | | | NR | | NR | | NR | | | NR | | |  | | | NR | | | NR | | | NR | |  | | | NR | | |
| 6. Were research personnel blinded to the study group during the study? | | | NR | | NR | |  | | | NR | | NR | | NR | | | NR | | |  | | | NR | | | NR | | | NR | | NR | | | NR | | |
| 7. Were outcome data complete without attrition or exclusion from analysis? | | |  | |  | |  | | |  | |  | |  | | |  | | |  | | |  | | |  | | |  | | NR | | |  | | |
| 8. Can we be confident in the exposure characterization? | | |  | |  | |  | | |  | |  | |  | | |  | | |  | | |  | | |  | | |  | |  | | |  | | |
| 9. Can we be confident in the outcome assessment (including blinding of assessors)? | | |  | |  | |  | | |  | |  | |  | | |  | | |  | | |  | | |  | | |  | | NR | | |  | |  |
| 10. Were all measured outcomes reported | | |  | |  | |  | | |  | |  | |  | | |  | | |  | | |  | | |  | | |  | |  | | |  | |  |
| 11. Were there no other potential threats to internal validity | | |  | |  | |  | | |  | |  | |  | | |  | | |  | | |  | | |  | | |  | |  | | |  | |  |
|  |  |  | |  | |  | |  |  | |  | |  | | |  | | |  | | |  | | |  | | |  | | | | |  |  |  |  |
|  |  |  | |  | |  | |  |  | |  | |  | | |  | | |  | | |  | | |  | | |  | | | | |  |  |  |  |
| Guideline |  |  | |  | |  | |  |  | |  | |  | | |  | | |  | | |  | | |  | | |  | | | | |  |  |  |  |
| Low |  |  | |  | |  | |  |  | |  | |  | | |  | | |  | | |  | | |  | | |  | | | | |  |  |  |  |
| Probably Low |  |  | |  | |  | |  |  | |  | |  | | |  | | |  | | |  | | |  | | |  | | | | |  |  |  |  |
| Probably high |  |  | |  | |  | |  |  | |  | |  | | |  | | |  | | |  | | |  | | |  | | | | |  |  |  |  |
| Definitely high |  |  | |  | |  | |  |  | |  | |  | | |  | | |  | | |  | | |  | | |  | | | | |  |  |  |  |
|  |  |  | |  | |  | |  |  | |  | |  | | |  | | |  | | |  | | |  | | |  | | | | |  |  |  |  |
|  |  |  | |  | |  | |  |  | |  | |  | | |  | | |  | | |  | | |  | | |  | | | | |  |  |  |  |
|  |  |  | |  | |  | |  |  | |  | |  | | |  | | |  | | |  | | |  | | |  | | | | |  |  |  |  |
|  |  |  | |  | |  | |  |  | |  | |  | | |  | | |  | | |  | | |  | | |  | | | | |  |  |  |  |
