# Supplementary material for: Systematic review on e-cigarette and its effects on weight gain and adipocytes
Source: PLoS One. 2022 Jul 5;17(7):e0270818. doi: 10.1371/journal.pone.0270818 (PMC9255744; doi:10.1371/journal.pone.0270818)
Supplement: Attachment — Submitted filename: Response_31.05.22.docx [file pone.0270818.s003.docx]

**EDITORIAL**

| Comments | Response |  |
| --- | --- | --- |
| 1. Please ensure that your manuscript meets PLOS ONE's style requirements, including those for file naming. | We have formatted the current manuscript according to the PLOS ONE format. | Title page  References |
| 2. We note that the grant information you provided in the ‘Funding Information’ and ‘Financial Disclosure’ sections do not match. | We have made the necessary amendments to both sections. |  |
| 3. In your Data Availability statement, you have not specified where the minimal data set underlying the results described in your manuscript can be found. We will update your Data Availability statement to reflect the information you provide in your cover letter. | We have updated the information on Data availability.  **Data Availability:** All relevant data are within the paper and its Supporting Information files |  |
| 4. Please review your reference list to ensure that it is complete and correct. | We have reviewed and edited the references to the best of our knowledge. | References |

**REVIEWER 1**

| Comments | Response |  |
| --- | --- | --- |
| As I said, I appreciate when the tables in reviews can make the data clearer. In this case they do, but I noticed some little issues with the table formatting (especially table 1).   a) Can you avoid the “jumping” words? That also in order to define the limits of every virtual column and avoid misunderstandings. b) Moreover, I would prefer if the author could choose an order for the points of the table 1 (for example, listing the papers following the year of publication). c) I suggest also to check every possible typing error (missing some capital letters). | Thank you for the comments.   a) We have formatted the columns to prevent ‘jumping’ of words. b) Table 1 was rearranged according to the publication year in descending order. Table 2 was rearranged according to type of study. c) We have checked and corrected the possible typos. | Table 1 Pg 10-12  Table 2 Pg 19-24 |
| The attention to details makes happier every reviewer: please, check the numbers at the bottom of the page. | We apologise for the mistake and have corrected the page numbers accordingly. |  |
| To better understand the difference between E-cigarettes (or other similar devices) and classic cigarettes, and how they could cause a different toxicity, I suggest to check a really recent multi-site study (Replica) conducted by the CoEHAR (Centre of Excellence for the Acceleration of Harm Reduction). It could be a nice reading, useful to enrich an already well written introduction. | Thank you very much for the comment. We have improved our introduction and added a couple of references. | Pg 4 |
